# Mutations outside the N-terminal part of *RBCK1* may cause polyglucosan body myopathy with immunological dysfunction: expanding the genotype–phenotype spectrum

**DOI:** 10.1007/s00415-017-8710-x

**Published:** 2017-12-19

**Authors:** Martin Krenn, Elisabeth Salzer, Ingrid Simonitsch-Klupp, Jakob Rath, Matias Wagner, Tobias B. Haack, Tim M. Strom, Anne Schänzer, Manfred W. Kilimann, Ralf L. J. Schmidt, Klaus G. Schmetterer, Alexander Zimprich, Kaan Boztug, Andreas Hahn, Fritz Zimprich

**Affiliations:** 1 Department of Neurology, Medical University of Vienna, Währinger Gürtel 18-20, 1090 Vienna, Austria; 2 CeMM Research Center for Molecular Medicine of the Austrian Academy of Sciences, Vienna, Austria; 3 Ludwig Boltzmann Institute for Rare and Undiagnosed Diseases, Vienna, Austria; 4 Institute of Clinical Pathology, Medical University of Vienna, Vienna, Austria; 5 Institute of Human Genetics, Technical University Munich, Munich, Germany; 6 Institute of Neurogenomics, Helmholtz Center Munich, Neuherberg, Germany; 7 Institute of Human Genetics, Helmholtz Center Munich, Neuherberg, Germany; 8 Institute of Medical Genetics and Applied Genomics, University of Tübingen, Tübingen, Germany; 9 Institute of Neuropathology, Justus Liebig University, Giessen, Germany; 10 Department of Otolaryngology, Göttingen University Medical School, Göttingen, Germany; 11 Department of Molecular Neurobiology, Max-Planck-Institute for Experimental Medicine, Göttingen, Germany; 12 Department of Laboratory Medicine, Medical University of Vienna, Vienna, Austria; 13 Department of Pediatrics and Adolescent Medicine, Medical University of Vienna, Vienna, Austria; 14 Department of Pediatrics, St. Anna Kinderspital and Children’s Cancer Research Institute, Medical University of Vienna, Vienna, Austria; 15 Department of Neuropediatrics, Justus Liebig University, Giessen, Germany

**Keywords:** Polyglucosan body myopathy, Glycogen storage disease, Cardiomyopathy, *RBCK1*, *HOIL*-*1*, Whole-exome sequencing

## Abstract

A subset of patients with polyglucosan body myopathy was found to have underlying mutations in the *RBCK1* gene. Affected patients may display diverse symptoms ranging from skeletal muscular weakness, cardiomyopathy to chronic autoinflammation and immunodeficiency. It was suggested that the exact localization of the mutation within the gene might be responsible for the specific phenotype, with N-terminal mutations causing severe immunological dysfunction and mutations in the middle or C-terminal part leading to a myopathy phenotype. We report the clinical, immunological and genetic findings of two unrelated individuals suffering from a childhood-onset *RBCK1*-asscociated disease caused by the same homozygous truncating mutation (NM_031229.2:c.896_899del, p.Glu299Valfs*46) in the middle part of the *RBCK1* gene. Our patients suffered from a myopathy with cardiac involvement, but in contrast to previous reports on mutations in this part of the gene, also displayed signs of autoinflammation and immunodeficiency. Our report suggests that *RBCK1* mutations at locations that were previously thought to lack immunological features may also present with immunological dysfunction later in the disease course. This notably broadens the genotype–phenotype correlation of *RBCK1*-related polyglucosan body myopathy.

## Introduction

Some glycogen storage diseases are characterized by the pathological accumulation of polyglucosan bodies, which consist of unspecific polysaccharides resistant to alpha-amylase digestion as a consequence of a defective glycogen metabolism [1]. Clinically, heart and skeletal muscles are predominantly affected due to their high physiological glycogen turnover [2, 3]. Homozygous or compound heterozygous mutations in the *RBCK1* gene were recently discovered to underlie a few cases with primary muscular involvement, a condition that was subsequently termed polyglucosan body myopathy 1 (PGMB1, MIM#615895) [4, 5]. From a functional point of view, the corresponding protein product HOIL-1 plays a crucial role in myogenesis and is enriched in fast-twitch glycolytic muscle fibres along with its interaction partners [6]. Accordingly, subjects with biallelic loss-of-function mutations resulting in HOIL-1 deficiency usually suffer from progressive muscular weakness and childhood- or juvenile-onset dilated cardiomyopathy, often necessitating heart transplantation at a young age [4, 5]. In addition to its function in muscle cells, HOIL-1 also constitutes an essential part of the so-called linear ubiquitination chain assembly complex (LUBAC), which regulates a variety of important NF-κB-dependent immune response mechanisms [7]. Affected subjects may simultaneously suffer from both chronic autoinflammation and immunodeficiency including recurring septicaemia [8]. The patients with *RBCK1* mutations reported so far vary considerably with respect to their leading clinical presentation (i.e., skeletal muscle, heart muscle, autoinflammation or immunodeficiency). The reason for this individual variability remains unclear, though it was hypothesized that the exact location of the variant within the gene might be a predictor for the predominant phenotype, with mutations in the N-terminal region of *RBCK1* primarily leading to immunological dysfunction and mutations in the middle or C-terminal parts rather resulting in a (cardio)myopathy phenotype [4].

Here, we report two unrelated individuals carrying the same homozygous mutation in the middle part of the *RBCK1* gene. Both presented clinically with a severe (cardio)myopathy and concomitantly with a comparatively mild, but clearly detectable immunological dysfunction. Our report sheds further light on the genotype–phenotype correlations of *RBCK1*-related diseases.

## Materials and methods

### Clinical evaluation

The clinical evaluation of affected individuals was performed at the Department of Neurology, Medical University of Vienna (Austria) and at the Paediatric Department of the Justus Liebig University of Giessen (Germany), respectively. This included a detailed medical history, a neurological examination, neurophysiological and routine laboratory investigations. Both subjects also underwent a skeletal and cardiac muscle biopsy. Peripheral nerve, liver and arterial vessel tissues were only investigated in Patient I.

### Whole-exome sequencing (WES) and data analysis

After obtaining written informed consent, blood samples were drawn and genomic DNA was extracted from leukocytes. WES was conducted at the Institute of Human Genetics, Technical University of Munich. Exomes were enriched in solution with *SureSelect Human All Exon Kit* (Agilent, 50 Mb V5) and DNA fragments were sequenced on an *Illumina HiSeq* *2500* system [9]. For data analysis, an autosomal recessive filter system for rare variants was used, followed by a screen for myopathy-related genes using keywords of the Online Mendelian Inheritance in Man (OMIM) database to narrow down the list of potentially causative variants. The study was approved by the local Ethics Committee of the Medical University of Vienna.

### Laboratory and immunological investigations

As part of routine diagnostics, antinuclear antibodies (ANA) and anti-neutrophil cytoplasmic antibodies (ANCA) were measured by indirect immunofluorescence using CE-certified diagnostic kits from *Aeskulides* (Wendelsheim, Germany) and *Euroimmun* (Lübeck, Germany), respectively. Antibodies to extractable nuclear antigens (ENA) were quantified using the Immunocap platform from *Phadia* (Uppsla, Sweden). Myositis-associated autoantibodies (including antibodies against AMA-M2, Jo-1, PM/Scl-100, PL-7, PL-12, Mi-2, Ku (p70/80), SRP, RibP) were measured using a diagnostic blot system from *Orgentec* (Mainz, Germany). Further, detailed immune phenotyping was performed. Histological investigation of tonsil tissue was additionally performed for Patient II.

## Results

### Clinical findings

#### Patient I

**Fig. 1:**
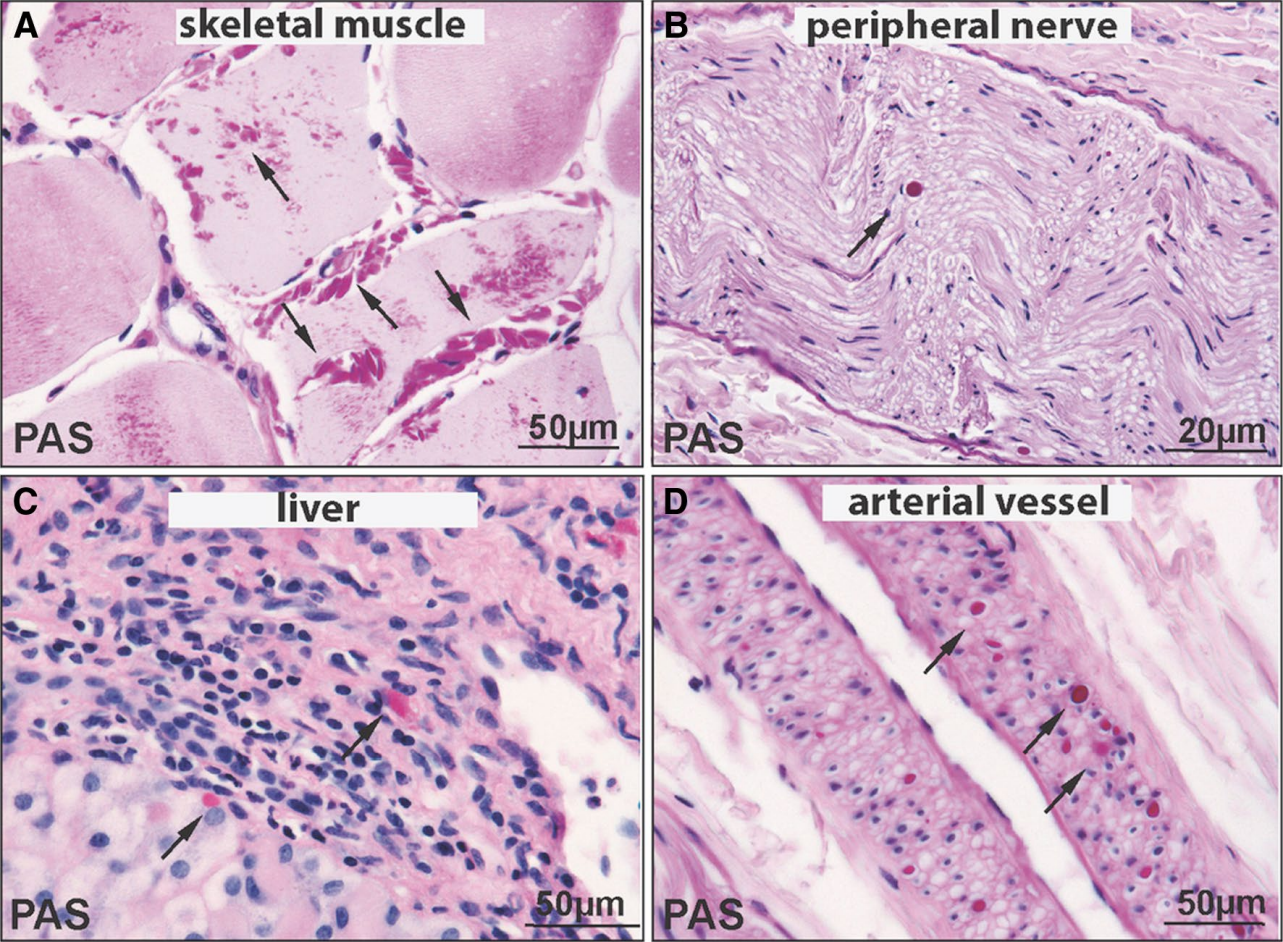
PAS-stained sections from Patient I demonstrating abundant PAS-positive polyglucosan bodies (arrows) in skeletal muscle (**a**), peripheral nerve (**b**), liver (**c**) and arterial vessel wall (**d**)

**Fig. 2:**
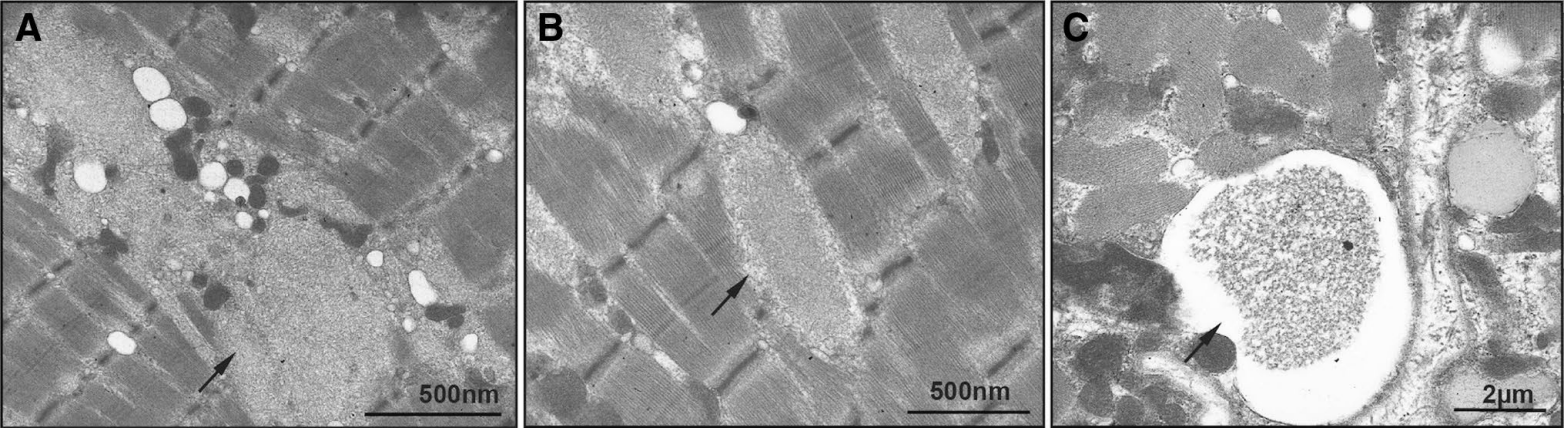
Ultrastructural analysis displaying polyglucosan bodies (arrows) in skeletal muscle fibres of Patient I causing myofibrillar disintegration (**a**, **b**), and subsarcolemmal accumulation of vacuoles filled with glycogen-storage material (arrow) (**c**)

This female patient was the third child of healthy Turkish parents. Her psychomotor development and cognitive status were reported to be normal. However, she experienced at least eight episodes of pneumonia and three episodes of unexplained fever persisting for several days. As she first presented with dyspnoea at the age of 14, echocardiography revealed a dilated cardiomyopathy with highly reduced left ventricular function. Consequently, she developed massive oedema, ascites and pleural effusions, requiring continuous diuretic treatment and an intercostal drain. Two months after the first presentation, a pacemaker had to be implanted due to multiple ventricular extrasystoles. A cardiac biopsy showed hypertrophic cardiac muscle cells containing enlarged vacuoles. An ultrasound of the abdomen revealed a hepatosplenomegaly. A neurological examination at age 15 displayed no facial weakness and generally normal muscle strength, except for a mild bilateral weakness of the pelvic girdle muscles with MRC (Medical Research Council) grade 4. Serum creatine kinase (CK) values were elevated up to 600 U/L (normal range < 180 U/L). Electromyography of deltoid, vastus lateralis and tibialis anterior muscles indicated a mild myopathy, whereas nerve conduction studies were normal. Abnormal accumulation of periodic acid-Schiff (PAS)-positive material, which is resistant to treatment with amylase, and polyglucosan bodies were found in skeletal muscle, peripheral nerve, liver and arterial vessel tissue, overall compatible with a diagnosis of glycogen storage disease (see Figs. 1 and 2).

At the age of 17 years, the condition worsened rapidly to NYHA (New York Heart Association) IV and the patient eventually succumbed to heart failure.

#### Patient II

This presently 33-year-old woman of Caucasian (Austrian) origin initially presented with a childhood-onset dilated cardiomyopathy, requiring heart transplantation at the age of 17 years. Histologically, basophilic degeneration and pronounced vacuoles were present in cardiac muscle cells. During childhood, the patient also suffered from relapsing episodes of viral (Herpes simplex) and bacterial (pneumonia, tonsillitis) infections. Additionally, in her 20s, she repeatedly displayed episodes of fever and rash, which could clinically be classified as acute febrile neutrophilic dermatosis (also referred to as Sweet’s Syndrome). This autoimmune condition promptly responded to systemic treatment with corticosteroids, but invariably relapsed as soon as the steroid dosage was reduced. At the age of 21, the patient developed a progressive muscular weakness affecting all four limbs, eventually rendering her wheelchair dependent 10 years later. A detailed neurological examination at the age of 32 documented a bilateral ptosis, a proximally pronounced weakness of the lower limbs (MRC 2, pelvic girdle) with relatively preserved strength in distal leg muscles (MRC 4). Upper limbs were also, but less severely, affected (MRC 4- in the shoulder girdle and MRC 4 + in distal muscles). Routine laboratory investigations showed a mild leucocytosis (13 G/L) under steroid treatment and fluctuating CK serum levels between 80 U/L and 1000 U/L (normal < 170 U/L). Electromyography of the quadriceps femoris muscle showed a myopathic pattern. Nerve conduction studies were normal. An MRI scan of the thigh muscles and the pelvic girdle confirmed a marked generalized muscular atrophy. A subsequent histological investigation of muscle tissue (vastus lateralis muscle) was inconclusive, merely showing fatty and fibrotic degeneration. The family history was generally unremarkable. Both parents (aged 72 and 67) and two siblings (aged 42 and 37) were alive and neither reported similar symptoms. Consanguinity was explicitly denied.

In Table 1, clinical details of both reported patients are summarized and compared to the previously published family carrying the same mutation.

**Table 1:** Clinical findings in patients I and II compared to the previously published cases BIII:1 and BIII:2 (one family) harbouring the same *RBCK1* mutation (c.896_899del) in a homozygous state [4]

	BIII:1 (Nilsson et al. [4])^a^	BIII:2 (Nilsson et al. [4])^a^	Patient I (this study)	Patient II (this study)
Gender	Female	Male	Female	Female
Age	24 (2013)	19 (2013)	Died at 17	32 (2017)
Age of onset	6	5	14	12
Initial presentation	Leg weakness	Difficulty running	Dyspnoea	Dyspnoea
Mobility	Walks without aid	Walks without aid	Walked without aid	Wheelchair
Facial weakness	None	None	None	Mild
Serum CK	X5	X6	X3	X6
Cardiomyopathy	DCM	DCM	DCM	DCM
Heart transplant	Age 14 years	Age 13 years	Not performed	Age 17 years
Autoinflammation	None	None	ANA mildly increased	Sweet’s syndrome
Immunodeficiency	None	None	Recurrent infections, unexplained fever	Recurrent bacterial and viral infections

^a^Family 10 in Table 2

*CK* creatine kinase, *DCM* dilated cardiomyopathy, *ANA* antinuclear antibodies

### Genetic findings

WES was first applied to Patient I in 2011. At that time, no underlying genetic cause could be identified. When WES was performed for Patient II in 2016 (after the publication of the first patients with *RBCK1*-related polyglucosan body myopathy), the homozygous frameshift variant NM_031229.2:c.896_899del, p.Glu299Valfs*46 in the gene *RBCK1* (MIM*610924) was detected as the most likely cause of the phenotype. As a consequence, the previously unclear case of Patient I (stored in the same in-house database) could be resolved in retrospect, showing the same *RBCK1* variant in a homozygous state. The variant is considered pathogenic according to the Human Gene Mutation Database (HGMD) (http://www.hgmd.cf.ac.uk/ac/index.php) and could only be found twice in a heterozygous state in 196,632 alleles of the Genome Aggregation Database (gnomAD) (http://gnomad.broadinstitute.org). Identity-by-state (IBS) analysis using next-generation sequencing data allows determining whether individuals are related [10]. Interestingly, both individuals reported in this article are not related to each other, which is indicated by an IBS of 0.06. Nevertheless, they share a common disease-associated haplotype of approximately 750 kb implying a founder effect.

Taken all together, a diagnosis of *RBCK1* deficiency-related polyglucosan storage disease could be established for both subjects on the basis of WES.

### Immunological findings

In Patient I, repetitive laboratory testing revealed mild lymphopenia with 3–4% of CD19 + cells (normal range: 6–25%) and low IgA levels, while IgG and IgM levels were within the normal range. In addition, autoantibody testing showed mildly elevated ANA titers in the range between 1:160 and 1:320, for which no particular aetiology could be identified. Overall, no specific autoimmune phenomena requiring treatment were observed in Patient I. Relapsing infections were treated with antibiotics if required.

Patient II underwent a tonsillectomy due to relapsing upper respiratory tract infections at the age of 15 years, i.e., before any immunosuppressive treatment was initiated. Histological analysis revealed an unusual, but unspecific non-necrotizing granulomatous inflammation. Routine tests for ANA, ANCA and antibodies to ENA as well as a myositis western blot were all negative at the age of 30 years. Additional lymphocyte function tests and immune phenotyping revealed no significant abnormalities. However, the patient was under oral treatment with corticosteroids at the time of testing.

## Discussion

In this report, we describe two new patients with pathogenic mutations in the *RBCK1* gene and thus expand the total number of known families with this condition in the literature from 12 to 14. We can confirm the few previous reports insofar as both a myopathy and an immunological phenotype are part of the clinical spectrum of *RBCK1*-associated disease.

Previous reports presented cases with a strong preponderance of either the immunological or the myopathic phenotype and it still remains unclear what exactly determines the clinical picture.

The patients described by Boisson et al. (all harbouring mutations in the N-terminal part of *RBCK1*) suffered from severe, childhood-onset immunological dysfunction leading to early death in infancy due to septicaemia [8]. In contrast, the majority of patients published by Nilsson et al. and Wang et al. carried mutations in the middle or C-terminal part of the gene and showed a later developing neuromuscular and cardiac involvement [4, 5].

**Fig. 3:**
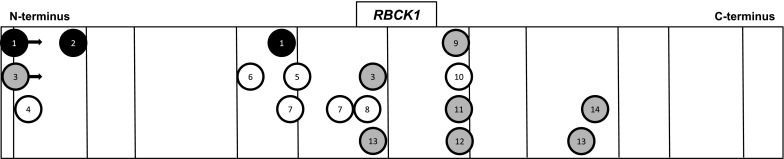
Schematic representation of published families with mutations in *RBCK1* (numbers according to Table 2). White circles represent a myopathy phenotype, grey circles represent myopathy with mild immunological dysfunction and black circles represent myopathy and severe immunodeficiency/autoimmunity. Black arrows indicate large deletions extending beyond the circles. Exon boundaries are indicated by vertical bars. Families with compound heterozygous states are represented twice

It was subsequently hypothesized that the nature and localization of the underlying mutation might predict the phenotype, with N-terminal mutations mainly causing immunological dysfunction. In contrast, variants in the middle- or C-terminal regions were presumed to predominantly cause cardiomyopathy and neuromuscular symptoms. Further, it was suggested that truncating variants might generally result in more severe phenotypes than missense mutations (see Table 2 and Fig. 3 for all reported families including main phenotype and variant localization) [4].

**Table 2:** Type and localization of mutations and main clinical characteristics of previously published families with *RBCK1*-related phenotypes (families sorted by localization of the mutations from N- to C-terminus)

Family	Mutation(s)^a^	Affected exons	Age at onset (years)	Myopathy/Cardiomyopathy	Immunodeficiency	Autoinflammation	Prognosis	References
1	c.ex1_ex4del	E1-4	<1	+	++	++	Died during childhood	Boisson et al. [8]
	p.Q185*	E5						
2	p.L41fs*7	E2	<1	+	++	++	Died during childhood	Boisson et al. [8]
3	p.E243Gfs*114	E6	4	++	+	+	Died at age 20	Nilsson et al. [4]
	c.ex1_ex4del	E1-4						
4	p.A18P	E2	Childhood	+	–	–	Alive at age 19	Nilsson et al. [4]
5	c.456 + 1G > C	E5/6 (intronic)	8	+	–	–	NA	Wang et al. [5]
6	p.R165Rfs*111	E5	9	++	–	–	Died at age 15	Nilsson et al. [4]
7	p.Q222*	E6	8	+	–	–	NA	Wang et al. [5]
	p.E190fs	E5						
8	p.A241Gfs*34	E6	Childhood	+	–	–	Alive at age 29	Nilsson et al. [4]
9	p.R298Rfs*40	E7	17	+	–	+	Alive at age 32	Nilsson et al. [4]
10	p.E299Vfs*18	E7	5, 6	++	–	–	Alive at age 19, 24	Nilsson et al. [4]
11	p.E299Vfs*46 ^**b**^	**E7**	**14**	**++**	**+**	**+**	**Died at age 17**	**This paper (Patient I)**
12	p.E299Vfs*46 ^**b**^	**E7**	**12**	**++**	**+**	**+**	**Alive at age 33**	**This paper (Patient II)**
13	p.E243*	E6	12, 16	++	–	+	Alive at age 47, 50	Nilsson et al. [4]
	p.N387S	E9						
14	p.R352*	E9	12	++	–	–	Alive at age 26	Nilsson et al. [4]

Patients reported in this paper are indicated in bold

*E* exon, *+* mild phenotype, *++* dominating phenotype, *NA* not applicable

^a^All reported mutations are biallelic (two listed mutations in case of compound heterozygous one listed mutation in case of homozygous state)

^b^Mutations of our reported families (11 and 12) refer to the RefSeq transcript NM_031229.2

The present characterization of two subjects with a homozygous mutation in the middle part (exon 7, RING domain) of the gene expands our still scanty knowledge on the genotype–phenotype correlation insofar as both patients developed both an immunological and a myopathic phenotype, although (cardio)myopathy was the dominant clinical feature in both cases. Additionally, the two (unrelated) individuals also suffered from recurrent bacterial and viral infections including severe and prolonged manifestations such as pneumonia. Patient II developed a dermatologic condition called Sweet’s Syndrome later in the disease course, which is currently considered to be an autoinflammatory disease [11]. Generalized erythema was previously described for N-terminal *RBCK1* mutations with immunological phenotypes by Boisson et al. [8]. Further, Patient II showed a non-necrotizing granulomatous reaction pattern of the tonsils, an uncommon, but unspecific histological finding suggestive of an exaggerated immune response [12, 13]. Interestingly, this phenomenon has previously been associated with an N-terminal deletion and a compound heterozygous frameshift variant in the middle part of *RBCK1* by Nilsson et al. (Family 3). Another individual (Family 13) suffered from sarcoidosis, which is also characterized by granulomatous inflammation. Further, type 1 diabetes (Family 13) and gluten intolerance (Family 9) were mentioned in the same publication [4]. We suggest that all these conditions may potentially represent a manifestation of autoimmune pathology related to mutations in *RBCK1*. In contrast, the patients of Nilsson et al. carrying the same variant as our patients (c.896_899del) were not reported to display any autoinflammation or immunodeficiency phenotype. However, we cannot exclude that subtle immunological symptoms either occurred later in the disease course or remained unreported due to a mild phenotype in these cases. In comparison, the disease onset in our cases was at a higher age (12 and 14 vs. 5 and 6 years) and rather dominated by cardiac symptoms than by muscular weakness. Ultimately, all four patients with this truncating mutation developed dilated cardiomyopathy either resulting in heart transplantation (3 subjects) or death due to heart failure (1 subject).

Considering all cases reported to date, it appears that pathogenic *RBCK1* variants appear to invariably cause a myopathy, but not necessarily immunological symptoms. So far, severe immunological phenotypes have only been reported for protein damaging N-terminal mutations, but milder immunological dysfunction is apparently not limited to this gene region (Fig. 3). A detailed review of the literature alongside with our newly reported cases shows that frameshift mutations beyond the N-terminus of *RBCK1* may lead to a combined phenotype including both myopathy and immunological dysfunction in single families. We are aware that truncating mutations may potentially be associated with nonsense-mediated decay, eventually resulting in the complete absence of a functioning protein [14]. This obviously limits any conclusions of the within-gene location of mutations on the phenotype. However, the fact that some reported frameshift mutations outside the N-terminal region (as in our patients) do not cause a predominant, severe immunological phenotype, suggests that some protein function is remaining in these cases, which would support the view that the phenotype is at least partly explained by the within-gene location of the mutation.

As our cases demonstrate, a precise molecular diagnosis might provide supporting information prior to invasive treatments such as heart transplant surgery or stem cell transplantation, which was reported as a therapeutic option by Boisson et al. in *RBCK1*-related cases with severe immunodeficiency [8]. For example, in Patient II, neuromuscular symptoms had first been attributed to a treatment with corticosteroids. Knowing the underlying genotype, the myopathy was much more likely to be part of the genetic syndrome rather than a side effect of medication. This again highlights the clinical usefulness of next-generation sequencing techniques in complex neurogenetic diseases [15–18].
